# Phase IV Drug Trials With a Canadian Site: A Comparison of Industry-Funded and Non-Industry-Funded Trials

**DOI:** 10.34172/ijhpm.2024.8239

**Published:** 2024-04-08

**Authors:** Joel Lexchin, Blue Miaoran Dong, Aravind Ramanathan, Marc-André Gagnon

**Affiliations:** ^1^School of Health Policy and Management, York University, Toronto, ON, Canada.; ^2^Department of Family and Community Medicine, University of Toronto, Toronto, ON, Canada.; ^3^School of Journalism and Communication, Carleton University, Ottawa, ON, Canada.; ^4^School of Public Policy & Administration, Carleton University, Ottawa, ON, Canada.

**Keywords:** Health Canada, Postmarket Trials, Phase IV Trials, Pharmaceutical Industry, ClinicalTrials.gov, Completed Trials

## Abstract

Recent regulatory reforms have favored expedited drug marketing and increased reliance on Phase IV clinical trials for safety and efficacy assurance. This study, utilizing ClinicalTrials.gov, assesses the characteristics of Phase IV trials, with at least one site in Canada, examining those funded by industry sponsors and those lacking industry funding. Additionally, it compares the publication status of industry-funded and non-industry-funded trials through a manual review of the medical literature. Between 2000 and 2022, 864 Phase IV trials were completed, with 480 (55.6%) receiving industry funding and 384 (44.4%) funded solely by non-industry sources. Industry-funded clinical trials were larger (mean 204 enrollees versus 70), more likely to be international (57.7% versus 9.6%) and reported results more promptly (1.21 years after completion versus 1.85 years), yet both types shared similar designs, outcomes, and completion times. Publication rates were 81.8% for industry-funded and 65.8% for non-industry-funded trials. The ClinicalTrials.gov registry displayed 48 inaccuracies in publication associations, raising concerns about its accuracy. Our findings underscore the existing institutional limitations in ensuring comprehensive reporting and publication of Phase IV trial results funded by both industry and non-industry sources.

## Background

 Before new medicines can be marketed they need to go through three phases of clinical testing to demonstrate efficacy for the indication(s) that they will be used to treat and to show that they are safe enough to be used.^1^ After medicines have been approved by Health Canada they sometimes undergo Phase IV testing. Phase IV studies are designed to gather information on issues such as the best way to use a drug and long-term benefits and risks.^2^ Commonly conducted studies include those dealing with safety issues and ones designed to support use under the approved indication, for example, mortality and morbidity studies, or epidemiological studies.^3^ Health Canada also treats the postmarket studies that are typically required to verify the clinical benefit of the drug when medicines are approved through its Notice of Compliance with conditions policy as Phase IV studies (Personal communication, Bureau of Policy, Science, and International Programs, March 22, 2023).

 Phase IV trials do not have to be approved by Health Canada as long as they are conducted within the parameters of the approved indication(s)^3^ and as a result protocols for these studies are not reviewed by Health Canada prior to the start of the trial. Nor is there a systematic and comprehensive collection of information contained within Health Canada’s clinical trials database that is designed to provide information about Canadian clinical trials involving human pharmaceutical and biological drugs.^4^

 Health Canada’s draft guidance from early 2023 encourages trial registration but does not mandate it,^5^ resulting in limited knowledge about the characteristics and quality of Phase IV studies with a site in Canada. In the context of a growing number of orphan drugs and niche medications, there has been a push by regulatory agencies to speed up their approval and rely on Phase IV clinical studies to confirm efficacy and safety. In this regard, the European Medicines Agency experimented with “adaptive licensing” and “adaptive pathways”^6^ while Canada is now moving towards “agile licensing.”^7^

 Industry funded phase IV trials may not contain all the necessary information to verify efficacy and safety. The German registry for Phase IV clinical trials showed that out of 558 industry-funded trials, no single adverse drug reaction report could be identified.^8^ However, because the results were not compared with non-industry-funded trials, it is unclear if the lack of information about safety applies equally as well to non-industry-funded trials.

 This study investigates the demographics of Phase IV trial participants in Canada, including gender, age, and enrollment numbers, along with trial factors such as funding source, trial completion time, and the duration from trial completion to results publication. The analysis compares these attributes between trials funded by industry sponsors and those with other funding sources, also examining the publication status of industry-funded versus non-industry-funded trials.

## Methods

###  ClinicalTrials.gov Trial Selection 

 The ClinicalTrials.gov database, widely recognized for its extensive repository of clinical trial information, was employed in this study due to its comprehensive nature containing information on over 470 000 studies in all 50 US states plus 222 countries and because it is specifically mentioned by Health Canada as a registration site.^3^ According to US legislation, trials have to be registered on ClinicalTrials.gov if they were commenced after September 27, 2007, are interventional, other than phase 1, study a Food and Drug Administration (FDA)-regulated drugs product and are produced in the United States or the clinical trial has a US FDA Investigational New Drug number.^9^

 Phase IV trials conducted in Canada or with a Canadian site were searched in ClinicalTrials.gov by a single investigator between March 12-15, 2023. The search focused exclusively on completed trials, considering the general understanding that non-completed trials are not typically expected to be published and if they are published will not contain complete results. The search for trials with partial or total industry funding used the check boxes and text fields in ClinicalTrials.gov as follows: Canada [country of origin] AND phase IV OR phase 4 [phase] AND industry [funder type] AND completed [recruitment] AND drug [intervention/treatment]. A similar search for trials with non-industry funding replaced “industry” with “NIH” OR “U.S. federal” OR “Other” in the funder type field. The search results were then downloaded as Excel files.

###  Publication Status

 The ClinicalTrials.gov database has a field that records publication status and the source of the publication for some trials. We rechecked publication status in cases where this field was blank and if a trial still lacked any documented publication in ClinicalTrials.gov, a search was undertaken between April 17, 2023 and May 25, 2023. During the course of this publication search, a comprehensive validation process was implemented to ascertain the presence or absence of any publication associated with the identified trial. Publications resulting from the trials were determined by inserting the ClinicalTrials.gov identification number into the search box on the National Library of Medicine (NLM) website (https://pubmed.ncbi.nlm.nih.gov). If there were no results, then the “Other IDs” were used if they were recorded in the files downloaded from ClinicalTrials.gov. Finally, the complete trial title was used as the search term. If there was a match or close match using trial title then the number of enrollees, trial location and date of the start of the trial were used to ensure that the trial shown on the NLM site was the same as the one registered on ClinicalTrials.gov.

 If searching on the NLM site was unsuccessful, then the ClinicalTrials.gov identification number, Other ID and title were sequentially searched through Google Scholar and the same method was used to determine if there was concordance between a publication identified on Google Scholar and the trial registered on ClinicalTrials.gov. If a publication was found, then the date it was published was recorded on the same Excel spreadsheet. If more than one publication was associated with a ClinicalTrials.gov identification number, an “Other ID” or the title then the one with the earliest publication date was chosen, unless the publication date was before the completion of the trial. In that case, the date of the first publication after the trial was finished was used. We only recorded details about publications after a trial was completed on the grounds that publications while the trial was in progress would not have the final results. If a trial was initially published as an Epub then that date was used.

 The presence of publications for industry-funded and non-industry-funded trials was independently searched separately by BMD (non-industry funded) and AR (industry funded). After each author had completed 10 searches, they were checked by a third author (JL). Once consensus about publication status was reached between JL and the other two, BMD and AR continued to search for publications for the remainder of the trials.

 The median time from study completion to publication is 14.5 to 30.8 months^10^ and the World Health Organization (WHO) recommends that trial results be published within 2 years of trial completion.^11^ Therefore, publication status was only assessed for trials completed up to May 31, 2021.

###  Data Analysis

 For trials with distinct funding sources, the following characteristics were computed: (*i*) percentage of trials with partial or complete industry funding versus other funding; (*ii*) percentage of trials conducted exclusively in Canada versus those spanning both Canada and international sites; (*iii*) percentage of trials with reported results; (*iv*) distribution of trial designs, including percentages of single arm, non-randomized and randomized trials; (*v*) total number of enrolled patients in each trial; (*vi*) duration in years between trial initiation and completion dates; and (*vii*) categorization of outcome measure (surrogate, clinical scale, clinical), determined from the “outcome measure” column in the ClinicalTrials.gov downloaded files. In instances where both surrogate and clinical or clinical scale outcomes were present, preference was accorded to the clinical/clinical scale outcome.

 In addition to trial characteristics the demographics of patients enrolled in the two differently funded types of trials were calculated: (*i*) percent of children (age less than 18), children and adults and adults/older adults and (*ii*) percent enrolling both sexes, males only and females only.

 Publication dates, start and completion dates in the ClinicalTrials.gov file were sometimes only given as month and year. In that case, the first day of the month was used. The time in years between completion date (up to May 31, 2021) and publication date was calculated along with the percentage published with each type of funding. A sensitivity analysis looking at the publication status of all registered trials including those completed after June 1, 2021 was also undertaken to see if including the additional trials changed the results.

 Trial characteristics, patient demographics, publication percentages, and time to publication were compared between industry and non-industry-funded trials using appropriate statistical tests (Chi-square or Mann-Whitney), with significance set at a two-sided *P* value of.05. The mean time to publication was computed using the time from the study completion date, as given in the ClinicalTrials.gov database, to the publication date.

 All calculations were done using Prism 9.5.1 (GraphPad Software, LLC).

## Results

 A single investigator searched ClinicalTrials.gov from March 12 to 15, 2023. Out of a total of 16 178 Phase IV trials registered there were 864 (5.3%) completed ones that were conducted in Canada or included a Canadian site; 480 (55.6%) with partial or complete industry funding and 384 (44.4%) with only non-industry funding. Start dates ranged from December 1, 1994 to February 25, 2022 and completion dates from October 1, 2000 to November 30, 2022. (Start dates were not listed for 5 industry-funded trials and 1 non-industry funded trial). Of those with industry funding, 336 (70%) were exclusively funded by industry and the remainder had a combination of industry and other funding. Although the search strategy specified completed trials there were 23 industry funded ones and 9 non-industry funded ones without a completion date recorded (Table S1 in Supplementary file 1 provides the complete data set used in this study).

###  Trial Characteristics

Table 1 presents the characteristics for trials with industry and non-industry funding. Industry funded trials were different from non-industry funded trials in a number of respects. They were significantly more likely to be based both in Canada and internationally compared to trials with non-industry funding that were usually conducted just in Canada (Chi-square, *P* < .0001). Industry-funded trials were also significantly more likely to have results reported (Chi-square, *P* < .0001) and the results were reported more rapidly (*P* = .0011, Mann-Whitney test). The median number of enrollees in industry-funded trials was 204 (interquartile range [IQR] 67, 550) compared to 70 (IQR 31, 181) for non-industry-funded trials (Mann-Whitney, *P* < .0001).

**Table 1:** Characteristics of Phase IV Cllinical Trials

**Metric **	**Type of Funding**	
	**Industry **	**Non-industry **
Sites	(n = 456)	(n = 384)
Canada only	193 (42.3%)	347 (90.4%)
Canada + international	263 (57.7%)	37 (9.6%)
Chi-square test	*P* < .0001	
Results	(n = 480)	(n = 384)
Present	263 (54.8%)	53 (13.8%)
Absent	217 (45.2%)	331 (86.2%)
Chi-square test	*P* < .0001	
Reporting results	(n = 263)	(n = 53)
Time in years (IQR)	1.21 (1.01, 1.81)	1.85 (1.13, 3.73)
Mann-Whitney test	*P* = .0011	
Enrollees	(n = 476)	(n = 382)
Number (IQR)	204 (67, 550)	70 (31, 181)
Mann-Whitney test	*P* < .0001	
Design	(n = 480)	(n = 383) [One study with cross-over design]
Single arm	67 (14.0%)	51 (13.3%)
Non-randomized	55 (11.5%)	26 (6.8%)
Randomized	356 (74.2%)	306 (79.9%)
Chi-square test	*P* = .0518	
Outcome	(n = 469)	(n = 378)
Clinical	153 (32.6%)	107 (28.3%)
Clinical scale	53 (11.3%)	48 (12.7%)
Surrogate	263 (56.1%)	223 (59.0%)
Chi-square test	*P* = .3829	
Study duration	(n = 457)	(n = 375)
Years from start to completion (IQR)	2.26 (1.42, 3.56)	2.59 (1.25, 3.98)
Mann-Whitney test	*P* = .4588	

Abbreviation: IQR, interquartile range.

 The three areas where the two types of trials were similar were in the design, distribution of the outcomes and the length of time from start to completion. Trial design was equally distributed between single arm, non-randomized and randomized (Chi-square, *P* = .0518); outcomes were equally distributed between clinical, clinical scales and surrogate (Chi-square, *P* = .3829); industry-funded-trials ran for 2.26 (IQR 1.42, 3.65) years and for non-industry-funded trials it was 2.59 (IQR 1.25, 3.98) years (Mann-Whitney, *P* = .4588).

###  Characteristics of Patients Enrolled in Trials

Table 2 shows that most trials were conducted in adults and older adults, both in the industry-funded group and in the non-industry funded group, 85.2% (409/480) and 82.8% (318/384), respectively, and the distribution between age groups was the same for trials with both types of funding (Chi-square, *P* = .135). Among 480 industry-funded trials, 91.5% enrolled both sexes, slightly surpassing the 85.4% observed in 384 non-industry-funded trials. The breakdown between the number of males and females was not given for trials with either type of funding. Overall, the sex distribution between the trials with the two types of funding was significantly different (Chi-square, *P* = .0062).

**Table 2:** Demographics of Patients Enrolled in Phase IV Trials

**Metric**	**Type of Funding**	
	**Industry**	**Non-industry**
Age	(n = 480)	(n = 384)
Children	23 (4.8%)	31 (8.1%)
Children + adults	48 (10.0%)	35 (9.1%)
Adults + older adults	409 (85.2%)	318 (82.8%)
Chi-square test	*P* = .136	
Gender	(n = 480)	(n = 384)
Males and females	439 (91.5%)	328 (85.4%)
Males only	27 (5.6%)	16 (4.2%)
Females only	24 (5.0%)	40 (10.4%)
Chi-square test	*P* = .0062	

###  Publication Status

 There were 433 industry-funded trials and 357 non-industry-funded trials that were completed before May 31, 2021. ClinicalTrials.gov listed 269 industry-funded trials and 247 non-industry-funded trials with linked publications. Twenty-three industry-funded publications and 25 non-industry funded publications were incorrectly linked by ClinicalTrials.gov to the wrong trials. Twelve industry-funded trials and 73 non-industry funded-trials that ClinicalTrials.gov listed were published before the trial start date and were not used. Finally, we were able to identify an additional 105 publications correctly associated with industry-funded trials and 84 publications correctly associated with non-industry-funded trials using either the NLM or Google Scholar. In total, 339 (81.8%) of industry-funded and 235 (65.8%) non-industry-funded trials that were completed before May 31, 2021 had publications (Table 3).

**Table 3:** Phase IV trials With Associated Publications

	**Industry-Funded Trials**	**Non-Industry-Funded Trials**
Trials identified by ClinicalTrials.gov	269	247
Trials with publications not associated with the trial	-23	-25
Trials with publication dates before trial completion	-12	-71
Additional trials identified using either NLM or Google Scholar	105	84
Total number of trials with publications	339	235

Abbreviation: NLM, National Library of Medicine.

 Table S2 in Supplementary file 2 provides a year-by-year and complete overview of the publication rates and time from trial completion to publication for industry-funded compared to non-industry-funded trials. Industry publication rates were higher than non-industry ones in 12 of the 19 years. Publication rates were the same in one year and in three years there were no non-industry-funded trials completed. Overall, industry-funded trials were no more likely to have been published compared to non-industry-funded trials (*P* = .1319, Chi-square test). Non-industry-funded trials were published more rapidly in 15 of the 18 years when time to publication could be compared between the two groups and over the entire time period were published more quickly – 1.50 years (IQR 0.83, 2.43) compared to industry funded ones – 2.00 years (IQR 1.31, 2.96) (*P* < .0001, Mann-Whitney test). There were no non-industry-funded trials completed in 2000-2003 that were published (Supplementary file 2, Table S2). A visual inspection of Supplementary Table S2 did not appear to show any changes in either the percent of trials being published or in publication times for either industry-funded or non-industry-funded trials post 2017 compared to earlier.

 The sensitivity analysis which included an additional 24 industry-funded trials, 11 with publications and 22 non-industry-funded trials, 12 with publications, that were completed after June 1, 2021 did not result in any statistically significant difference in publication rates or time to publication between industry-funded and non-industry-funded trials.

## Discussion

 In our analysis of 864 Phase IV clinical trials with at least one Canadian site registered on ClinicalTrials.gov, we observed that industry-funded trials, comprising 55.6% of the total, were larger, more often had international sites, had a higher proportion with published results and those publications appeared more promptly compared to the 44.4% non-industry-funded trials. Despite differences in funding sources, both types of trials showed similar design characteristics, outcome measures, and completion times. Another study focusing on Canadian trials on ClinicalTrials.gov similarly noted superior reporting in industry-funded trials compared to those from academia.^12^ The higher proportion of trials with industry-funding that we found is in concordance with the 63% figure for industry-funded trials that Bourgeois et al found^13^ but in contrast with Hoffmann and colleagues’ report of a higher proportion of Phase IV trials originating from academia.^14^

 The Food and Drug Administration Amendments Act of 2007 mandates sponsors of applicable trials to report results on ClinicalTrials.gov within 1 year of completion, with a starting date for the initial trials subject to this requirement of January 2018. Other studies have suggested that this change may have led to increased compliance in reporting for industry-funded trials compared to trials funded by other sources^15^ but our study did not support this finding.

 Poorer reporting by researchers with non-industry funding compared to researchers with industry funding has been shown in a number of other publications,^15-17^ although this conclusion is not universal.^18^ Zwierzyna and colleagues also found that studies with industrial funding were substantially larger than non-industry funded ones and more likely to include international locations^17^ as did Bourgeois et al.^13^ These results probably reflect the greater level of financial and human resources that are available to large pharmaceutical companies and possibly the difference in the motivation for carrying out the trials; non-industry-funded trials may be motivated by academic interests whereas industry-funded trials are likely to be motivated by commercial reasons.

 Our ability to examine patient characteristics in Phase IV trials by funding status was limited by the availability of this type of information in ClinicalTrials.gov, however we found that while the age range in trials was similar, the sex breakdown was not. We are not aware of results about patient characteristics being published before and the similarities and differences should be further investigated.

 The rate of publication for non-industry-funded trials in our study was 65.8% compared to 81.8% for industry-funded trials, although this difference was not statistically significant. Our publication rate for non-industry funded studies is in line with the 68% rate reported by Ross and colleagues^19^ and with the 64% rate for all phases of clinical trials regardless of funding.^15^ Although Hoffmann and coworkers also reported a higher publication rate for industry funded Phase IV trials compared to those with other types of funding, our rates were considerably higher than theirs for both groups.^14^ Other studies have reported the reverse of what we found – higher publication rates for non-industry/non-government funded trials compared to industry funded ones.^13,20^

 Median times from trial completion to publication were 2.00 years for those with industry funding and 1.50 years for those with other types of funding, a statistically significant difference. Our time from completion to publication for non-industry-funded trials was considerably shorter than the 23 months (1.92 years) that others found for National Institute of Health funded trials.^19^ Although industry-funded trials reported results more quickly in ClinicalTrials.gov than did non-industry-funded ones, they were slower to publish their results in journals. Recording results first in clinical trials registries might be a priority for industry if trials were being carried out for regulatory purposes, but except for the minority that were undertaken to satisfy their Notice of Compliance with conditions, Phase IV trials in our study were being carried out for other reasons.

 The reasons for publication and non-publication of industry and non-industry-funded trials are likely to be different. While industry has an incentive to hide results showing their drugs in a less flattering light, academics who conduct the large majority of non-industry-funded trials might more easily abandon a trial with negative results in order to focus on studies that could boost their academic career.^21,22^ Publication rates for industry-sponsored trials may also be higher, since those trials are more likely to stress positive results and positive conclusions than trials with any other type of funding.^23^

 The accuracy of some of the information in ClinicalTrials.gov registry is questioned by our finding that 48 publications that were listed were not correctly associated with the trials that were registered. In addition, ClinicalTrials.gov also includes pre-research articles published by the same group of investigators who subsequently undertake the trial. In our case this occurred 83 times. While these publications may contain important information for the study, they will not contain study results.

 We did not compare the quality of reporting in ClinicalTrials.gov and in publications, but Hartung et al found discrepancies between the two and were not able to conclude which was more accurate,^24^ whereas Riveros et al concluded that reporting was more complete in publications than in ClinicalTrials.gov.^25^ However, both ClinicalTrials.gov and journal publications are inferior to clinical study reports in the quality and quantity of information reported.^26,27^

###  Limitations

 The results only apply to clinical trials and not other types of studies, eg, observational ones and only to trials that were registered in ClinicalTrials.gov and had at least one Canadian site. Trials in other stages of recruitment, eg, not yet recruiting, active but not recruiting, suspended were not included. When identifying publications, we lacked the resources to analyze the content of each article in order to verify their alignment with the information reported in ClinicalTrials.gov. Finally, there are the limitations of the ClinicalTrials.gov database; for example, there is no information about the cost of the clinical trials nor about any conflict-of-interest of the investigators.

## Conclusion

 The importance of ClinicalTrials.gov as a comprehensive resource of information about clinical trials of all types cannot be understated, but it is essential for the platform to prioritize meticulousness in order to ensure accurate and reliable reporting. Non-publication or the lack of accuracy about publication of Phase IV clinical trials remains too common. This situation is particularly acute in Canada since there is no other way to track Phase IV trials.

 Industry sponsored trials appear to be more compliant with registration and reporting standards than non-industry ones, but the accuracy of the information in ClinicalTrials.gov for both types of trials has not been systematically examined and additional research in this area is necessary.

 There has been significant pressure calling for regulatory reforms in favor of faster marketing of drugs and stronger reliance on Phase IV clinical trials for ensuring the safety and efficacy of drugs. However, our main conclusion based on the results of our study is that we still do not have the necessary institutional capacity to ensure comprehensive reporting and publication of results for a substantial number of these trials.

## Ethical issues

 All data were publicly available and ethics approval was not required. No patients were involved in this study.

## Competing interests

 Joel Lexchin received payments for writing briefs on the role of promotion in generating prescriptions for two legal firms between 2019 to 2023. He is a member of the Board of Canadian Doctors for Medicare. He receives royalties from University of Toronto Press and James Lorimer & Co. Ltd. for books he has written. Blue Miaoran Dong and Aravind Ramanathan report no conflicts-of-interest. Marc-André Gagnon received payment for organizing a workshop on models of pharmaceutical production for *Association des pharmaciens des établissements de santé du Québec. *

## Funding

 This study was supported by an Insight Grant from the Social Sciences and Humanities Research Council of Canada (SSHRC) (#435-2021-0715). The funders had no role in the design, conduct, or publication of the content.

## Supplementary files

Supplementary file 1 contains Table S1.

Supplementary file 2 contains Table S2.
