# Supplementary material for: Phase IV Drug Trials With a Canadian Site: A Comparison of Industry-Funded and Non-Industry-Funded Trials
Source: Int J Health Policy Manag. 2024 Apr 8;13:8239. doi: 10.34172/ijhpm.2024.8239 (PMC11607594; doi:10.34172/ijhpm.2024.8239)
Supplement: Supplementary file 2 — contains Table S2. [file ijhpm-13-8239-s002.pdf]

Article title: Phase IV Drug Trials With a Canadian Site: A Comparison of Industry-Funded and Non-IndustryFunded Trials

Journal name: International Journal of Health Policy and Management (IJHPM)

Authors' information: Joel Lexchin[1,2]*, Blue Miaoran Dong[3], Aravind Ramanathan[4], Marc-André Gagnon[4]

[1]School of Health Policy and Management, York University, Toronto, ON, Canada.

[2]Department of Family and Community Medicine, University of Toronto, Toronto, ON, Canada.

[3]School of Journalism and Communication, Carleton University, Ottawa, ON, Canada. [4]School of Public Policy & Administration, Carleton University, Ottawa, ON, Canada.

***Correspondence to:** Joel Lexchin; Email: jlexchin@yorku.ca

Citation: Lexchin J, Dong BM, Ramanathan A, Gagnon MA. Phase IV drug trials with a Canadian site: a comparison of industry-fundedand non-industry-funded trials.Int J Health Policy Manag. 2024;13:8239. doi:10.34172/ijhpm.2024.8239

Supplementary file 2

Supplementary Table S2: Phase IV trial completion and publication status by funding status and year

**Year completed**	**Industry-funded**			**Non-industry-funded**		
	**Number completed**	**Number published**	**Median time to publication (years, interquartile range)**	**Number completed**	**Number published**	**Median time to publication (years, interquartile range)**
**2000**	1	1 (100%)	8.84 (8.84, 8.84)	0	0	NA
**2001**	0	0	NA	0	0	NA
**2002**	4	4 (100%)	2.63 (1.84, 4.37)	3	0 (0%)	NA

**2003**	2	2 (100%)	1.22 (0.93, 1.50)	0	0	NA
**2004**	13	10 (76.9%)	2.88 (1.54, 4.91)	2	2 (100%)	7.35 (2.09, 12.6)
**2005**	16	10 (62.5%)	2.63 (1.72, 3.76)	8	5 (62.5%)	1.83 (0.53, 4.74)
**2006**	26	22 (84.6%)	2.94 (1.55, 4.61)	8	5 (62.5%)	1.46 (1.25, 7.38)
**2007**	27	20 (74.1%)	2.53 (2.05, 5.39)	16	12 (75.0%)	2.21 (0.91, 3.74)
**2008**	35	29 (82.9%)	2.97 (1.97, 4.18)	15	6 (40.0%)	1.12 (0.94, 1.94)
**2009**	24	18 (75.0%)	1.69 (1.16, 2.78)	14	9 (64.3%)	1.50 (0.76, 8.76)
**2010**	25	25(100%)	2.36 (1.33, 3.16)	20	10 (50.0%)	1.62 (0.77, 2.29)
**2011**	22	17 (77.3%)	2.00 (1.48, 2.01)	22	18 (81.8%)	1.45 (0.71, 3.44)
**2012**	27	20 (74.1%)	1.88 (1.51, 3.00)	20	15 (75.0%)	2.00 (1.42, 3.07)
**2013**	33	28 (84.8%)	1.91 (1.07, 2.81)	20	12 (60.0%)	1.49 (0.56, 2.69)
**2014**	17	13 (76.5%)	2.34 (1.48, 3.12)	29	19 (65.5%)	1.76 (1.25, 3.50)
**2015**	22	19 (86.4%)	1.49 (0.81, 2.44)	32	23 (71.9%)	1.59 (1.11, 3.39)
**2016**	27	21 (77.8%)	1.36 (0.79, 2.70)	28	16 (57.1%)	1.10 (0.43, 1.80)
**2017**	38	31 (81.6%)	1.67 (0.83, 2.39)	22	16 (72.7%)	1.78 (1.02, 3.68)

**2018**	30	23 (76.7%)	1.59 (1.09, 1.95)	34	23 (67.6%)	1.13 (0.78, 2.01)
**2019**	24	15 (62.5%)	2.02 (1.52, 2.35)	29	19 (65.5%)	1.50 (0.67, 2.09)
**2020**	14	6 (42.9%)	1.17 (1.00, 1.60	29	21 (72.4%)	1.12 (0.72, 1.79)
**2021**	6	5 (83.3%)	1.90 (1.88, 2.16)	6	4 (66.7%)	1.02 (0.50, 1.36)
**Total**	**433**	**339 (81.8%)**†	**2.00 (1.31, 2.96)**‡	**357**	**235 (65.8%)**	**1.50 (0.83, 2.43)**

*To May 31, 2021

†Compared to non-industry-funded trials, $p = 0.1319$, Chi-square test

‡Compared to non-industry-funded trials, $p < 0.0001$, Mann-Whitney test
